# Investigating the Usability of a Multidimensional Metaverse Rehabilitation Platform for Survivors of Colorectal Cancer: Mixed Research Design Experiment

**DOI:** 10.2196/63543

**Published:** 2026-07-31

**Authors:** Yuru Hu, Zheng Ruan, Huan Peng, Zhongmou Huang, Xinhui Yu, Lu Cai, Yao Xiao, Bo Chen, Xinyu Huang, Qiufen Sun, Haiyan Zhuo, Qu Shen

**Affiliations:** 1 Department of Colorectal Surgery, The First Affiliated Hospital of Xiamen University, School of Medicine, Xiamen University, Xiamen, China, 86 17274967315; 2 Xingtai Medical College, Xingtai, China; 3 Teaching and Research Section of Clinical Nursing, Xiangya Hospital of Central South University, Changsha, China

**Keywords:** metaverse, multidimensional rehabilitation, colorectal cancer survivors, radiation therapy, survivor, colorectal cancer, chemotherapy, quality of life, sexual dysfunction, irregular bowel, health education, diet, exercise, interventions, intervention

## Abstract

**Background:**

Metaverse technology is transforming health care, offering a wide range of applications in telemedicine, clinical care, education, mental health, and physical well-being.

**Objective:**

This study aimed to explore the usability of a multidimensional metaverse rehabilitation platform for survivors of colorectal cancer. The platform integrates exercise training, dietary guidance, mental health education, and behavioral management.

**Methods:**

A mixed research design was used. Survivors of colorectal cancer and their family members were recruited at the affiliated hospital to use the Metaverse multimodal rehabilitation platform. The platform’s performance was quantitatively evaluated using Post-Study System Usability Questionnaires (PSSUQs) and System Usability Scales (SUSs). The average scores and SDs for each dimension were calculated, and the results were depicted using radar charts and violin plots. Additionally, correlation analysis was concurrently used to investigate score variations across diverse dimensions concerning different demographic factors. Structured questions were used to conduct qualitative interviews with participants to gather their opinions, suggestions, and perceived benefits regarding the platform.

**Results:**

The data from a total of 18 participants were ultimately included, with an average age of 47 (SD 3.73) years. The overall SUS score was 65 (SD 4.157), and the average total score of PSSUQ was 80 (median 90, IQR 20). No statistically significant differences (*P*>.05) were observed in the overall scores and dimension scores of the SUS and PSSUQ across gender, age, and educational level distributions. The radar chart illustrates that the learnability score for the metaverse platform was the lowest (mean 61.11, IQR 5.08), while the Interaction Quality score was the highest (median 86.67, IQR 27). The results of the qualitative interviews indicated that users were generally highly satisfied with the platform’s content, finding it comprehensive, diverse in format, and convenient to use. However, the platform’s operation was not yet sufficiently streamlined, and its learnability still needs improvement. The metaverse multidimensional rehabilitation platform demonstrates clinical usability and holds potential to enhance the quality of life for survivors.

**Conclusions:**

The platform demonstrated strong clinical usability and received high ratings for several parameters. Learnability, usability, and satisfaction levels were found to be satisfactory, with no significant correlations with demographic factors.

## Introduction

After undergoing treatments, such as surgery, radiation therapy, and chemotherapy, survivors of colorectal cancer often face a range of physiological, psychological, and social challenges that significantly impact their quality of life [1,2]. Studies have shown that in the year following the end of treatment, survivors of colorectal cancer experience various persistent symptoms and functional impairments, including diarrhea, constipation, irregular bowel movements, urinary issues, and sexual dysfunction. Additionally, fear of disease recurrence, anxiety, and depression are common psychological issues among this group during the course of their illness [3]. Hence, there is a growing need to promote healthy behaviors and survivorship rehabilitation among survivors of colorectal cancer. However, current rehabilitation interventions in China predominantly focus on health education, offering postoperative dietary and exercise guidance. Due to this singular approach and the lack of comprehensive, targeted rehabilitation content, consistently improving patient outcomes poses a challenge [4].

The concept of “multidimensional rehabilitation” was first proposed by van Weert et al [5], who emphasized that multidimensional rehabilitation should encompass education, physical and psychological interventions, exercise training, dietary advice, and other aspects aimed at addressing cancer-related functional impairments and improving patients’ quality of life. In studying the effects of multidimensional rehabilitation, Mewes et al [6] defined it as a combination of rehabilitation interventions targeting 2 or more dimensions of the International Classification of Functioning, Disability, and Health. These interventions typically involve various exercises, cognitive behavioral therapy, psychological therapy (including counseling and symptom self-management), and strategies to aid patients in returning to work. Within the biopsychosocial medical model, the concept of “multidimensional rehabilitation” based on the International Classification of Functioning, Disability, and Health framework has been formally established. Compared with single-dimensional interventions, the combined effects of multidimensional rehabilitation often yield more significant results. Multidimensional rehabilitation programs primarily focusing on physical activity and dietary interventions significantly aid survivors of colorectal cancer in maintaining long-term health and quality of life [7]. Yun et al [8] conducted a web-based health guidance program involving physical activities, dietary adjustments, and posttraumatic growth behaviors, resulting in improved health behaviors among survivors of colorectal cancer. Hawkes et al [9] provided health behavior guidance and supervision via phone calls, effectively enhancing the dietary habits, physical activity levels, and BMI of survivors of colorectal cancer.

In the current medical landscape, interventions leveraging the internet and artificial intelligence (AI) have emerged as promising strategies for promoting healthy behaviors and lifestyles, widely used in clinical intervention research [10,11]. To some extent, these technologies can address the shortcomings of the current rehabilitation management model for survivors of colorectal cancer. However, existing intelligent intervention methods face some issues, such as small-scale, irregular processes, and a lack of comprehensiveness. Furthermore, existing multidimensional rehabilitation platforms cannot fully meet the needs of survivors of colorectal cancer for multidimensional rehabilitation. Therefore, constructing a multidimensional rehabilitation platform with diversity, immersion, and sustainability is the key to addressing these problems.

Metaverse refers to a virtual space that is parallel to and independent of the real world [12]. By integrating various technologies, such as the internet, computers, AI, electronic games, and displays, a highly realistic digital virtual world is constructed. The metaverse has been widely applied in various fields, including remote health care, clinical care, education, mental health, and physical health [13]. It supports remote health care and can overcome geographical limitations [14]. Integrating the metaverse into health care has the potential to significantly impact clinical practice and improve human well-being [15].

The metaverse spans a wide range of fields, including computer science, social sciences, architecture, arts and humanities, business and economics, management, education, law, medicine, and the environment [16]. In the medical field, metaverse technologies such as 5G, virtual reality, augmented reality, mixed reality, AI, blockchain, big data, and haptic internet are actively being introduced, demonstrating significant potential in patient diagnosis, treatment, and rehabilitation. An increasing number of medical institutions use virtual reality technology for adjunctive therapy and remote rehabilitation [17]. This metaverse also shows great potential in the field of rehabilitation medicine [18]. For example, abroad, clinical application programs, such as rehabilitation, help physical therapists and medical workers to communicate efficiently with patients, and rehabilitation management systems enable real-time monitoring of patient rehabilitation training status, with results uploaded to the cloud for analysis and guidance by rehabilitation experts. Additionally, the metaverse is used to create communication platforms for patients with cancer and their families, assisting patients in improving the discomfort caused by the disease and enhancing their quality of life [19].

In conclusion, the metaverse has broad application prospects in the medical field, including in disease prevention, health management, diagnosis, and treatment. Applying the metaverse to the field of rehabilitation medicine is highly forward-thinking and innovative. With the development of technologies, such as AI and the internet of things, the metaverse is poised to bring new opportunities to rehabilitation medicine, improve the quality of life of survivors of colorectal cancer, and reduce the incidence of complications. Additionally, as a new form of participation and multidimensional interactive scene, the metaverse provides possibilities for a multidimensional rehabilitation model for survivors of colorectal cancer, aiding in the transition from “passive health care” to “active health.”

In recent years, mobile health (mHealth) apps have played an increasingly prominent role in disease management, health promotion, and public health. mHealth provides health monitoring, intervention, and management services through mobile terminals, such as smartphones and wearable devices. However, the usage rates are not optimistic, and the actual effectiveness heavily relies on the usability of the apps. This often results in high-cost apps failing to fully realize their expected scientific and clinical value. Studies indicate that many apps suffer from poor user experiences, low acceptance rates, and poor user retention due to issues like complex interfaces and cumbersome operations, ultimately affecting the achievement of intervention goals [20,21]. Particularly for the older adult population, poor usability is a critical factor hindering their adoption of mobile medical technologies [22]. An mHealth app with a user-friendly interface can enhance user satisfaction, increase accessibility, and reduce the risk of harm [23]. Therefore, improving usability is not only a technological optimization requirement but also a necessary condition to ensure user health benefits.

Usability is defined as the “ability of specific users to achieve goals effectively, efficiently, and satisfactorily through a product in a given environment” and has become a core criterion for evaluating digital health technologies [24]. International guidelines emphasize that usability testing is an indispensable part of health technology development [25]. Before using health technology as a health intervention measure, it is crucial to ensure that the technology is appropriately designed to meet the needs of end users. Conducting usability testing on mHealth apps holds significant value; well-designed products can enhance user satisfaction, increase willingness for continued use, improve user experience, enhance user retention, and better cater to the needs of different user groups (such as the disabled and older adults), bridging the digital divide and promoting health equity [26].

In conclusion, the metaverse has broad application prospects in the medical field, including in disease prevention, health management, diagnosis, and treatment. Applying the metaverse to the field of rehabilitation medicine is highly forward-thinking and innovative. With the development of technologies, such as AI and the internet of things, the metaverse is poised to bring new opportunities to rehabilitation medicine, improve the quality of life of survivors of colorectal cancer, and reduce the incidence of complications. Additionally, as a new form of participation and multidimensional interactive scene, the metaverse provides possibilities for a multidimensional rehabilitation model for survivors of colorectal cancer, aiding in the transition from “passive healthcare” to “active health.”

## Methods

### Study Design

Based on a preliminary investigation of the home-based rehabilitation needs of survivors of colorectal cancer, we developed a Meta-Universe Multidimensional Rehabilitation Platform. This platform serves both educational and health-promotion functions. By integrating various guidelines and existing intervention practices and after expert validation, we formulated a Meta-Universe multidimensional rehabilitation program tailored to survivors of colorectal cancer. This 4-week intervention program was divided into 3 parts each week—objectives, practice, and knowledge sharing. We aimed for this 4-week intervention program to have a positive impact on health promotion. Additionally, weekly knowledge-sharing sessions serve educational purposes. Subsequently, the entire program will be implemented on the Meta-Universe platform to design and develop the Meta-Universe Multidimensional Rehabilitation Platform.

Following the design of the Meta-Universe Multidimensional Rehabilitation Platform, qualitative interviews were conducted to optimize the platform, followed by usability studies. This study primarily reports the results of the usability research.

### Instrument Design and Development

The design of the Meta-Universe Multidimensional Rehabilitation Platform follows the steps outlined in Garrett’s user experience elements model, encompassing 5 layers—strategic, scope, structural, framework, and presentation [27].

*Strategic layer*: This involves defining the positioning goals of the meta-universal Multidimensional Rehabilitation platform. The platform targets survivors of colorectal cancer and their families after curative surgery. It primarily serves as a platform for survivors and their families to engage in integrated learning and communication, encompassing goal management, practice, knowledge, and interaction.

*Scope layer*: This layer focuses on the content design of the meta-universal Multidimensional Rehabilitation platform. The main content areas included physical rehabilitation, dietary and nutritional interventions, mental health education, and behavior management.

*Structural layer*: This step primarily involves an information framework. Within the Meta-Universe, a virtual environment is created to allow users to access rehabilitation resources, participate in activities, and interact with other participants. The structural framework is as follows.

*Main lobby*: Serving as the primary entrance and reception area of the platform, a central area was designated for project introductions and access points to various rehabilitation learning rooms. The lobby’s background music comprises gentle, soothing tunes to calm users’ minds, alleviate anxiety, tension, and negative emotions, and foster a sense of warmth and hope for the future.

- Rehabilitation learning rooms: These specialized areas facilitate learning, where users can engage in weekly rehabilitation goals, practices, and knowledge sharing. In total, 4 learning rooms were established according to the intervention plan and sequentially opened weekly. Users must complete the objectives of each week to progress to the next. Each learning room features sections for weekly objectives, practices, knowledge sharing, assessments, and goal completion checklists. In addition, guided tour services led by virtual robots are provided. These virtual assistants offer 24-hour guided tours and learning services to support and guide users throughout their learning journey. Users can engage in simultaneous online learning, communication, voice, and video interactions within rehabilitation learning rooms equipped with sofas and tables to create a conducive learning and communication atmosphere.
- Multipurpose conference room: This space serves as a versatile area for user discussions and interactions. It includes a large screen for hosting lectures related to family rehabilitation and user experience sharing activities. Message boards are set up for users to make public comments, along with bulletin boards for event announcements and knowledge updates. The conference room aimed to facilitate communication among participants and knowledge dissemination.

*Presentation layer*: This involves perceptual design, including page layout, color schemes, and sensory experiences, to create high-fidelity prototypes. Additionally, incorporating audio, video, and other multisensory design methods enhances the user experience (providing voice reading functions for both text and graphics). These measures deliver a sleek and engaging interface design for users.

The overall layout of the platform is depicted in Multimedia Appendix 1.

Before using the Meta-Universe Multidimensional Rehabilitation Platform, users will receive an instructional video explaining how to use it. When using the platform, users were required to follow a predetermined timeline for learning. Users must enter the corresponding room for each week’s learning; once the tasks for a week are completed, the room for the next week will unlock, gradually revealing the content for the third and fourth weeks. Completed rooms remain accessible for continuous learning. The ideal outcome of using the platform is for users to acquire knowledge of dietary habits, exercise routines, behavior management, and emotional regulation in daily life, develop self-management skills, and apply these practices effectively.

### Procedure

After the Meta-Universe Multidimensional Rehabilitation Platform was designed, we assembled a platform optimization team comprising health care professionals, survivors of colorectal cancer, and volunteer family members. This team was primarily responsible for ensuring the platform’s internal usability. External usability research was conducted once internal feedback had been provided. External usability research involved inviting survivors of colorectal cancer and their family members to use the meta-universal Multidimensional Rehabilitation platform. The duration of use was personalized based on each participant’s circumstances, allowing them to use the platform until they felt that they had fully understood and mastered its functionalities. Subsequently, the usability of the platform was assessed using relevant metrics, and user experience suggestions and feedback were collected.

After users were confirmed to participate, they were instructed and demonstrated by the researchers on how to operate the platform, following which the users commenced the trial. The trial was entirely self-directed, with no researcher involvement during the process; however, users could contact the researchers at any time if they had any questions. Considering the portability during the trial and the platform’s potential for future scalability, AR headphones were not used. This ensured that users could access the platform anytime and anywhere.

### Study Participants

The study was conducted from February 1, 2024, to April 1, 2024. Participants were recruited offline. Patients from the Department of Colorectal Surgery and Oncology at the Xiamen University Affiliated Hospital and Hunan Xiangya Hospital were selected. Those who met the inclusion criteria among survivors of colorectal cancer were contacted through face-to-face interviews or phone calls to confirm their willingness to participate in the study. Once the patients and their family members agreed to participate, the study participants met the inclusion and exclusion criteria.

Patients who underwent curative surgery for colorectal cancer and received adjuvant therapy, aged 18 years or older but younger than 75 years, not pregnant, willing to participate in the study, and able to use a mobile phone were included. Exclusion criteria included patients with multiple cancers, those with special dietary requirements, and individuals with cognitive impairments that could affect the use of the multidimensional rehabilitation platform.

Inclusion criteria for family members were family members of patients who underwent curative surgery for colorectal cancer and received adjuvant therapy, aged 18 years or older but younger than 75 years, willing to participate in the study, and able to use a mobile phone. Individuals with cognitive impairments that could affect the use of multidimensional rehabilitation platforms were excluded.

Purposeful sampling was implemented, with a small sample size (n<14) sufficient to reflect usability [28]. A total of 18 survivors of colorectal cancer and their family members were included. According to the relationship curve between the number of participants and the percentage of identified issues, 10 patients could identify 80% of usability issues, and 15 participants were adequate to identify 100% of usability issues [29]. Therefore, the minimum sample size for this study ranged from 10 to 15 individuals.

### Measures

The Methods section primarily involved questionnaire assessments, including basic information, a usability questionnaire, and 5 open-ended questions. The general information collected included name, age, level of education, role (patient or caregiver), and other relevant details.

The usability questionnaire assessment used 2 tools—the Post-Study System Usability Questionnaire (PSSUQ; version 3) and the System Usability Scale (SUS). The PSSUQ questionnaire consists of 3 dimensions (usefulness, information quality, and interface quality) and 1 overall evaluation item, totaling 16 items [30]. Each item was rated on a scale of “strongly disagree” to “strongly agree” with a range of 1‐5 points. To facilitate comparisons across dimensions, scores for each dimension were converted to a percentage scale, with higher scores indicating greater user satisfaction. The SUS was used to evaluate user experience using a Likert 5-point scale method with 10 questions. Among these questions, 1, 3, 5, 7, and 9 were positively phrased, whereas 2, 4, 6, 8, and 10 were negatively phrased. The validity of this scale is 0.85 [31]. SUS scores were calculated on a percentage scale, where a score above 70 indicated a “good” level, and above 50 indicated an “okay” level.

Additionally, the questionnaire included 5 open-ended questions, with the researchers responsible for recording the participants’ responses. The questions were as follows:

1. What is your overall impression of using this platform?

2. How beneficial is this platform for self-management and symptom management, and what are its advantages?

3. Which features of this platform are easier to use?

4. What shortcomings do you think exist in this platform?

5. How beneficial is this platform for self-management and symptom management, and what are its advantages?

### Data Collection

After patients or their family members have tried the platform, both parties will agree on a time and location for a face-to-face or remote assessment. The collection of intervention data will be the responsibility of the researcher. Participants can choose to fill out the questionnaire themselves or opt for one-on-one questioning by the researcher. Throughout the process, if participants have any questions about any item, the researcher will provide answers promptly to ensure the accuracy of the responses.

Following the completion of questionnaire data collection, interview data collection will commence. The interviews will be fully recorded, and the researcher will ask questions one by one according to the interview outline. If participants do not have clear answers to certain questions, the researcher will provide appropriate neutral guidance to elicit the participants’ true feelings. If participants are unable or unwilling to participate in oral interviews, they can also fill out the answers themselves based on the interview outline.

### Statistical Analysis

*Quantitative data analysis*: Continuous data will be presented as mean (SD) along with the range of values (minimum and maximum), while categorical data will be expressed as percentages. The Mann-Whitney *U* test and ANOVA will be used to investigate differences in the total and dimension scores of the SUS and PSSUQ scales based on gender, age, and educational level distributions. For data visualization, a violin plot will be used to display all data, while radar charts will visually represent the scores of each dimension and the total score of the usability scale.

*Interview data analysis*: Within 24 hours of the completion of the interviews, the interview content will be transcribed verbatim into text format. The content of the interviews will be carefully read, important information will be extracted and analyzed, and keywords and phrases that repeatedly appear will be recorded. All content and viewpoints will be summarized; similar viewpoints will be identified and organized into different themes.

### Ethical Considerations

Ethical approval was obtained from the Xiamen University School of Medical Research Ethics Board (approval XDYX202308K52). All methods were performed in accordance with relevant guidelines and regulations. The study adhered to the principles outlined in the Declaration of Helsinki and was conducted under continuous supervision of the ethics committee. Informed consent was obtained from all the participants. The researchers provided a written explanation of the study’s purpose and methods to the participants, emphasizing their voluntary participation and freedom to withdraw from the study at any time, without facing any consequences. Furthermore, it was clarified that the information provided would be used solely for research purposes and not for any other purpose. To protect participant privacy and confidentiality, all personal identifiers were removed or anonymized in the data collection, storage, and analysis processes. All confidential information and research data were securely stored and only accessible to authorized researchers involved in this study. No financial or other forms of compensation were provided to the participants in this study.

## Results

### Participant Characteristics

Recruitment involved 21 patients and their family members, with 3 individuals excluded (1 did not provide informed consent, 1 was older than 75 years old, and 1 selected the same option on the SUS scale). Ultimately, 18 participants were included in this analysis. Refer to Table 1 for detailed information about the participants.

**Table 1.** Demographic information of the participants (N=18).

Characteristics	Participants, n (%)
Sex	
Male	12 (66.67)
Female	6 (33.33)
Age (y)	
Average value	47 (3.73)
18-44	8 (44.44)
45-59	6 (33.33)
60-74	4 (22.22)
Highest completed education	
Junior high school or below	5 (27.78)
High school or technical secondary school	5 (27.78)
College or associate college	7 (38.89)
Master’s degree or above	1 (5.56)
Identity	
Survivors of colorectal cancer	10 (55.56)
Caregivers	8 (44.44)

### Usability Scale Analysis

The SUS and PSSUQ scores were converted to a percentage scale, with the specific scores presented in Table 2. The overall average and average scores for each dimension of both the scales exceeded 50. There were no significant differences (*P*>.05) in the overall scores and dimension scores of the SUS and PSSUQ based on gender, age, and educational level distributions. For detailed information, please refer to Tables 3 and 4.

**Table 2.** Usability testing scores (N=18).

Usability indicators	Statistical value	SD/IR	95% CI
SUS^a^_score, mean (SD)	65 (4.157)	4.157	56.23-73.77
Learnability, mean (SD)	61.11 (5.086)	5.086	50.38-71.84
Effectiveness, mean (SD)	66.67 (4.434)	4.434	57.31-76.02
Satisfaction, mean (SD)	66.67 (4.261)	4.261	57.68-75.66
PSSUQ^b^-sum, median (IQR)	80 (80-100)	20 (80-100）	80-100
SysQual^c^, mean (SD)	84.44 (2.681)	2.681	78.79-90.1
InfoQual^d^, mean (SD)	82.04 (3.213)	3.213	75.26-88.82
IntQual^e^, median (IQR)	86.67 (73.3-100)	27 (73.3-100)	73.3-100

a SUS: System Usability Scale.

b PSSUQ: Post-Study System Usability Questionnaire.

c SysQual: System Quality.

d InfoQual: Information Quality.

e IntQual: Interaction Quality.

**Table 3.** The distribution of SUS^a^ scores in different demographics.

Variable	n	SUS total			Learnability			Effectiveness			Satisfaction		
		Mean (SD)	*t* test (*df*) or *F* test (*df*)	*P* value	Mean (SD)	*t* test (*df*) or *F* test (*df*)	*P* value	Mean (SD)	*t* test (*df*) or *F* test (*df*)	*P* value	Mean (SD)	*t* test (*df*) or *F* test (*df*)	*P* value
Identity			−1.96 (16)^b^	.07		−1.82 (16)^b^	.08		−1.591 (16)^b^	.13		−1.874 (16)^b^	.80
Patient	10	58.25 (15.14)			53.33 (17.66)			60.63 (18.17)			60 (16.1)		
Dependents	8	73.44 (17.72)			70.83 (23.15)			74.22 (17.82)			75 (17.82)		
Sex			0.28 (16)^b^	.24		−0.56 (16)^b^	.58		0.65 (16)^b^	.52		0.68 (16)^b^	.51
Male	12	65.83 (15.75)			59.03 (19.61)			68.75 (16.21)			68.75 (16.71)		
Female	6	63.33 (22.51)			65.28 (26.57)			62.5 (24.37)			62.5 (21.57)		
Age (y)			1.02 (2,15)^c^	.38		4.55 (2,15)^c^	.03		0.32 (2,15)^c^	.73		0.36 (2,15)^c^	.70
18-44	8	71.56 (20.53)			73.96 (18.6)			70.31 (23.33)			70.83 (23.15)		
45-59	6	58.75 (13.58)			44.44 (19.48)			65.63 (12.96)			63.89 (11.39)		
60-74	4	61.25 (16.14)			60.42 (14.23)			60.94 (19.35)			62.5 (17.35)		
Years of schooling (y)			1.14 (2,15)^c^	.35		3.23 (2,15)^c^	.07		0.59 (2,15)^c^	.56		0.52 (2,15)^c^	.60
<9	5	57 (15.95)			51.67 (19.89)			58.75 (17.46)			60 (16.03)		
11-12	5	62.5 (12.5)			50 (19.55)			68.75 (11.69)			66.67 (10.21)		
>12	8	71.56 (20.53)			73.96 (18.6)			70.31 (23.33)			70.83 (23.15)		

a SUS: System Usability Scale.

b *t* test.

c *F* test.

**Table 4.** The distribution of PSSUQ^a^ scores in different demographics.

Variable	n	SysQual^b^			InfoQual^c^			IntQual^d^			PSSUQ total		
		Mean (SD)	*t* test (*df*) or *F* test (*df*)	*P* value	Mean (SD)	*t* test (*df*) or *F* test (*df*)	*P* value	Median (IQR)	*t* test (*df*) or *F* test (*df*)	*P* value	Median (IQR)	*t* test (*df*) or *F* test (*df*)	*P* value
Identity			−2.01^e^	.06		−0.24^e^	.82		—^f^	.57^g^		—	.46^g^
Patient	10	80.00 (10.19)			81.33 (14.16)			86.67 (70-98.34)			80 (70-95)		
Dependents	8	90.00 (10.84)			82.92 (13.85)			93.33 (75-100)			90 (80-100)		
Sex			0.29^e^	.78		1.06^e^	.30		—	.89^g^		—	.82^g^
Male	12	85.00 (10.87)			84.45 (12.66)			86.67 (73.33-100)			80.00 (80-100)		
Female	6	83.33 (13.34)			77.22 (15.41)			90.00 (70-98.34)			80.00 (70-90)		
Age (y)			1.37^h^	.28		2.00^h^	.17		—	.52^g^		—	.54^g^
18-44	8	85.42 (13.45)			77.08 (15.37)			86.67 (68.35-98.33)			80.00 (65-100)		
45-59	6	88.34 (8.37)			90.56 (8.54)			93.34 (83.34-100)			90.00 (80-100)		
60-74	4	76.67 (9.03)			79.17 (12.58)			83.34 (70-96.67)			80.00 (65-95)		
Years of schooling (y)			0.14^h^	.87		1.16^h^	.34		—	.22^g^		—	.38^g^
<9	5	82.00 (13.25)			83.33 (11.06)			80.00 (70-93.34)			80.00 (70-90.00)		
11‐12	5	85.33 (6.91)			88.67 (12.16)			100.00 (87.67-100)			100.00 (80-100)		
>12	8	85.42 (13.45)			77.08 (15.37)			86.67 (65.00-95.00)			80.00 (60-85)		

a PSSUQ: Post-Study System Usability Questionnaire.

b SysQual: System Quality.

c InfoQual: Information Quality.

d IntQual: Interaction Quality.

e *t* test.

f Not applicable.

g The data did not follow a normal distribution, used the rank sum test.

h *F* test.

### Radar Chart Analysis

In this study, we used the average and median values of each dimension of the SUS and PSSUQ (using the mean for normally distributed data and the median for nonnormally distributed data) to create radar charts and compare the relationships and trends between different variables. These variables include Learnability, Effectiveness, Satisfaction, System Quality (SysQual), Information Quality (InfoQual), and Interaction Quality (IntQual), which represent the 6 characteristics of the metaverse platform. Each variable is represented on a different axis, forming a closed polygonal area. The scale on each axis represents the range of the values for each variable.

**Figure 1.**
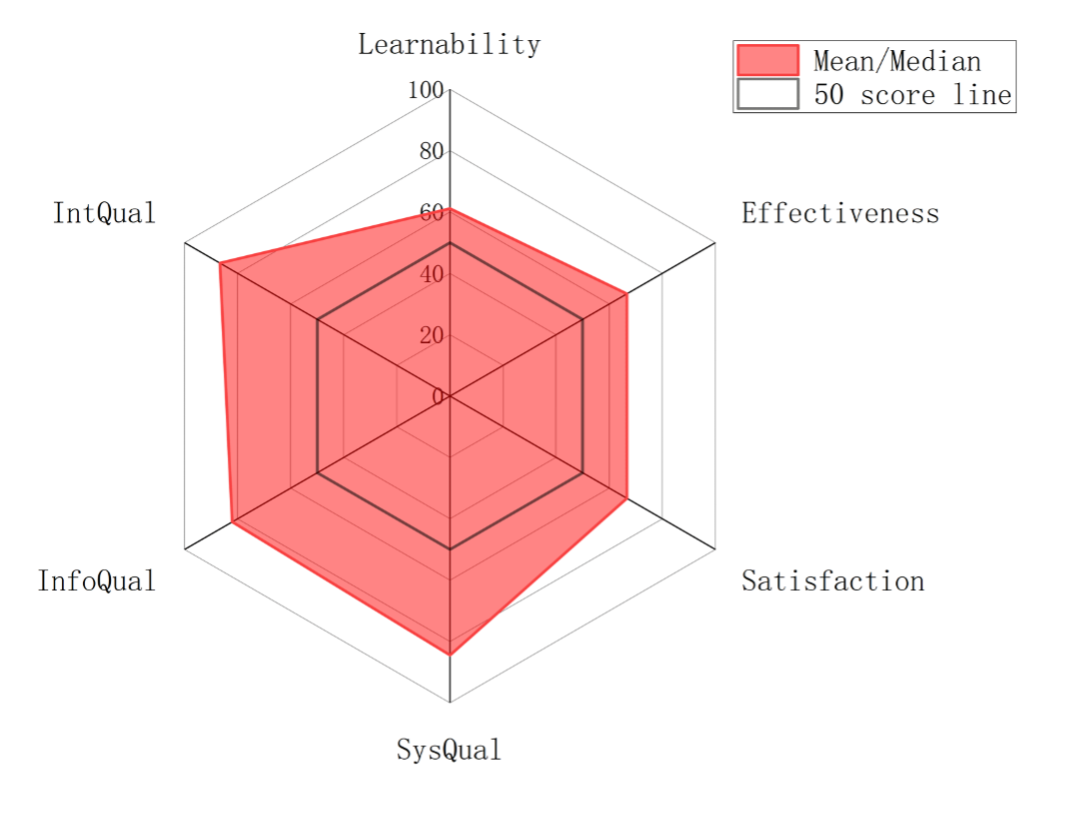
Usability testing scores radar chart.

From the radar chart, it is evident that the learnability score for the metaverse platform was the lowest (mean 61.11, SD 5.09, 95% CI 50.38-71.84), whereas the IntQual score was the highest (median 86.67, IQR 73.3-100). Refer to Figure 1 for more detailed information.

### Violin Plot Analysis

**Figure 2.**
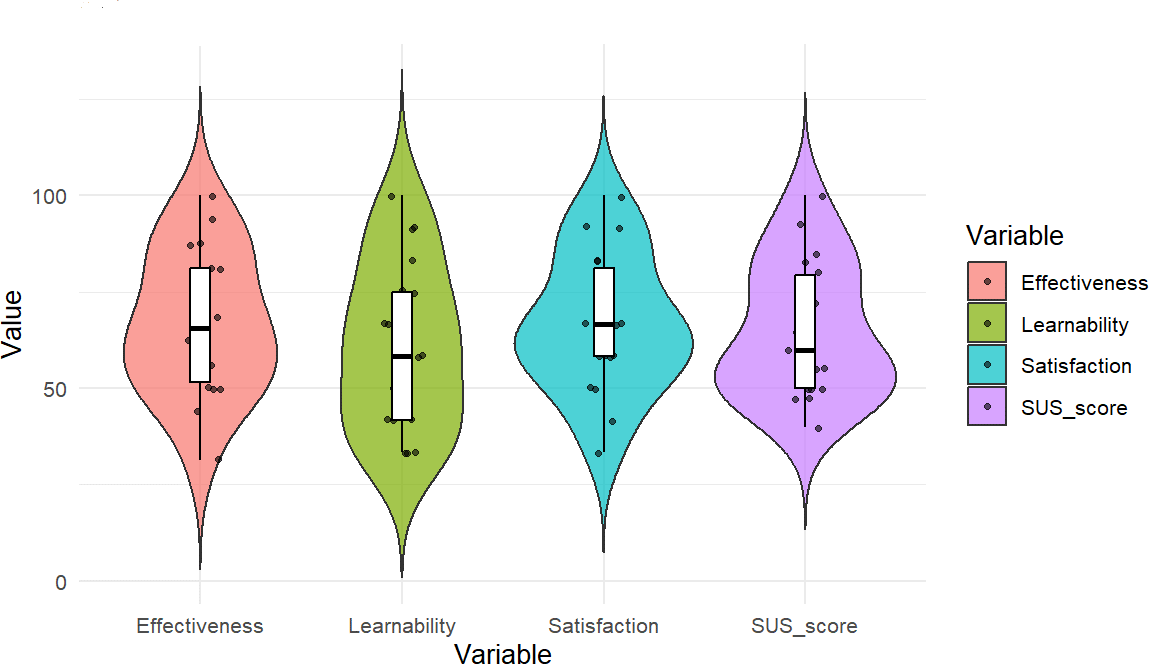
Violin plot of SUS scores.

Violin plots were used to display the distribution of scores. From the results of the violin plots, it can be observed that the distribution of the SUS total scores is relatively concentrated in the range of 50‐60, showing distinct peaks. The distribution of the SUS total scores ranged from 40 to 100. In the various dimensions of the SUS, the maximum value for effectiveness was 100, and the minimum value was 31. For learnability, the maximum value was 100 and the minimum was 33. The maximum satisfaction score was 100, with a minimum of 33. The distribution of learnability was concentrated below 50, whereas the scores for the other 2 dimensions were concentrated above 50. The scores for satisfaction and usability were concentrated between 50 and 75, above the OK line. Refer to Figure 2 for more detailed information.

**Figure 3.**
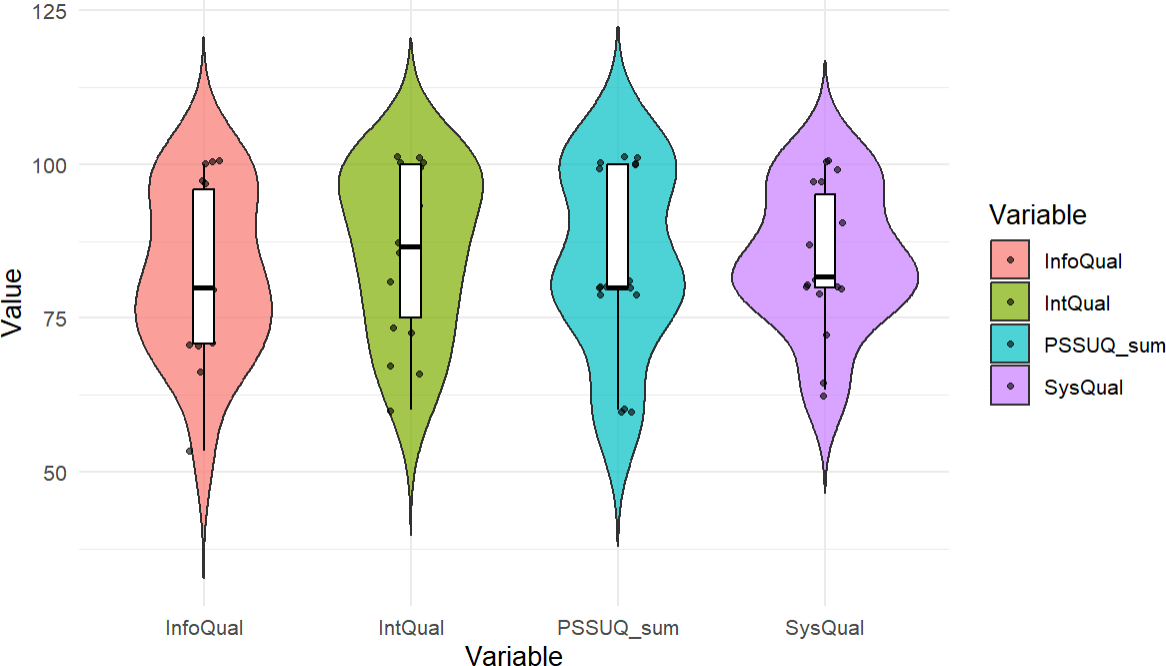
Violin plot of Post-Study System Usability Questionnaire scores.

The distribution of the total PSSUQ scores ranged from 60 to 100. In the various dimensions of the PSSUQ, the maximum value for InfoQual was 100 and the minimum value was 53. For IntQual, the maximum and minimum values are 100 and 60, respectively. SusQual had a maximum value of 100 and a minimum of 63. There are 2 concentration areas for the PSSUQ total scores, at 100 and 75‐87.5 points; InfoQual scores are concentrated around 100 and 75 points; IntQual scores are relatively concentrated between 95 and 100; and SysQual scores are mostly concentrated between 75 and 87.5, with another concentration area at 100 points. Refer to Figure 3 for more detailed information.

### Open-Ended Question Results

Interview data from 16 participants were collected, and the interview data for each participant is numbered from N1 to N16. A total of 4 main themes were identified based on the interview titles and content, and keywords were extracted for analysis.

#### Theme 1: Platform Evaluation

Keywords extracted fall into 3 categories—positive evaluation, neutral evaluation (including neutral evaluations containing needs), and negative evaluation. Each category’s keywords and their frequencies are as follows:

1. Positive Evaluation
  Excellent (13 times): eg, “Excellent.”
  Convenient/practical (12 times): eg, “Convenient,” “Practical,” “Relevant,” “Clear at a glance,” and “Easy to operate.”
  Useful/beneficial (10 times): Direct mentions of “Useful,” “Helpful,” and “Guiding significance.”
  Comprehensive/professional Content (8 times): eg, “Comprehensive content,” “Rich information,” and “Professional.”
  Interesting/advanced (5 times): eg, “Interesting like a game,” “Looks very advanced,” “High-end,” and “Advanced stuff.”
  Considerate/user-friendly (4 times): eg, “Considerate,” “Voice function is convenient,” and “Automatic playback is suitable for the elderly.”
2. Neutral Evaluation
  Average (4 times): eg, “Average, some good some not so good” and “Average.”
  Need guidance/optimization (6 times): eg, “Add beginner’s guide,” “Directory,” “Signage,” and “Optimize operation.”
  Age adaptation issues (2 times): “Elderly people may not know how to use” and “Automatic playback is more suitable for the elderly.”
3. Negative Evaluation
  Slow loading/lagging (8 times): eg, “Slow loading,” “Slow response,” “Takes too long to load,” and “Too laggy.”
  Complex operation/getting lost (7 times): eg, “Complex operation,” “Getting lost,” “Stuck in a corner,” and “Interface not intuitive enough.”
  Average (4 times): eg, “Average, some good some not so good” and “Average.”

#### Theme 2: Platform Advantages

Keywords extracted include comprehensive and professional content, practical multimedia functions, easy operation, interesting and innovative features.

*Found it useful, quite a lot of content, quite a few videos, don’t have to strain to read, I don’t like reading text stuff anyway. Will recommend it to others.*

[N1]

*Using this platform feels convenient, the information is quite comprehensive. Feels quite interesting, like a game, quite convenient.*

[N1]

*Feels pretty good, content is quite extensive, there are videos too, looks very advanced.*

[N2]

*Has videos, pictures and voice, not bad, sometimes can’t see the words clearly, the voice is good. Feels useful, like being in class, there will be a lot of detailed guidance, it’s useful if you find time to look at it.*

[N2]

*Overall, this platform is still practical, the content is quite comprehensive, looks quite professional, after all, it was developed by professionals, but I feel the content needs constant updating. For example, the app I’m using, Mi Jian, is very good, there is one-on-one communication with professionals, helping to read diagnostic reports, and giving advice, the response is very quick, this is something you can learn from.*

[N3]

*The overall user experience of this platform is very good, whether it’s exercise, diet, or mental health, it covers everything.*

[N4]

*The features are very practical, for example, with pictures, when I click, there’s actually voice, scared me! This can be noted, it’s too sudden, sound comes on as soon as you click, but this feature is good, you don’t have to read, you can listen while doing other things.*

[N4]

*Pictures can play, videos, all very practical.*

[N5]

*Pretty good, can learn some knowledge, the platform is quite considerate.*

[N6]

*Useful. After using it, taking care of patients will be much easier.*

[N6]

*The design is quite clear, easy to use, very convenient, and the operation is also very easy.*

[N7]

*Pictures, videos and such are very useful. Can learn some knowledge that is useful for diseases.*

[N8]

*Useful, has guiding significance for patients needing rehabilitation knowledge.*

[N9]

*Text comes with a reading function, suitable for a wide range of people.*

[N9]

*Good, I think a lot of things are quite practical.*

[N10]

*Useful, now at least know how to eat and exercise.*

[N10]

*Useful, there are a lot of things, quite comprehensive, basically whatever you want to see is there.*

[N11]

*(Features) are all good, but at first when I didn’t understand some things, it was a bit confusing, like the arrow on the ground, didn’t know what it was for at first, then realized.*

[N11]

*Useful, can help us learn a lot of knowledge.*

[N13]

*I think the information provided by this platform is quite rich, especially about psychology and behavior, very practical.*

[N14]

*I think the information provided by this platform is quite rich, especially about psychology and behavior, very practical.*

[N14]

#### Theme 3: Platform Shortcomings

Keywords extracted include lack of content, difficulty in operation, lack of interactivity, insufficient personalized service, lack of prompts, not concise, information overload, and difficulty in screening.

*Sometimes loading is a bit slow, hope it can be improved.*

[N1]

*The response is a bit slow, takes a long time to load, if you’re not patient you might exit, if the internet speed is not good, you might exit too. Sometimes when walking it’s a bit dizzying, a bit too flexible, got stuck in a corner and can’t see other places, can’t get out, feels lost inside. Not very easy to operate, need to be more familiar to operate.*

[N2]

*Not enough content, lack of interaction and personalized service, just like I said before, Mi Jian does it very well, you can download and take a look. Although the annual membership fee is not cheap, but it’s worth it!*

[N3]

*Can be more concise, more operational prompts, at the beginning it’s too troublesome, don’t know where to go, it’s better after watching the operation video, but still easy to get lost, stuck in a corner.*

[N5]

*However, sometimes the operation is a bit complex.*

[N5]

*More intelligent, the elderly don’t know how to use it.*

[N8]

*The screen is a bit laggy, guidance is not straightforward, room stickers are too simple.*

[N9]

*Using it is quite laggy, beginners need teaching for entry.*

[N9]

*The platform’s information classification is quite clear, but sometimes there’s too much information, it’s a bit difficult to choose, hope it can be more streamlined.*

[N10]

*But actually it’s not easy to find content, if you want to look at a specific category, this platform can’t search, have to look one by one.*

[N11]

*Entering this platform is a bit slow.*

[N12]

*But some functions are not very intuitive, especially in terms of exercise and physical activity, diet and nutrition, need to spend time exploring. Hope user operation convenience can be optimized.*

[N16]

*Too laggy, loading takes a long time.*

[N16]

#### Theme 4: Suggestions and Recommendations

Keywords extracted include (1) adding product recommendations; (2) setting different modes based on age, automatic playback for the older adults; and (3) adding beginner’s guidance. Most participants expressed no opinions or suggestions.

*It’s best to combine long-term use with product recommendations, recommend some products and services, spend a bit of money, for example, what to do with low white blood cells, need to supplement protein, at this time can have some reliable products.*

[N3]

*Set different modes for different ages, for the elderly it plays automatically, no need to click.*

[N8]

*Add beginner’s guidance, reading materials can be explained in the form of animation and text, the homogeneity of the survey questions is serious, adjustments need to be made according to the current room content.*

[N9]

## Discussion

### Principal Findings

Metaverse technology has been widely applied in various fields, such as telemedicine, clinical care, education, mental health, and physical well-being [32]. It supports remote health care, overcomes geographical limitations, and brings new and challenging opportunities to the health care sector [14]. An increasing number of medical institutions are beginning to adopt virtual reality technology for adjunct therapy and remote rehabilitation services [17]. The Metaverse multidimensional rehabilitation platform developed in this study for survivors of colorectal cancer covers exercise training, dietary guidance, psychological health education, and behavior management. It also includes a 4-week self-management intervention program aimed at helping survivors of colorectal cancer adopt healthy lifestyles, promote healthy behaviors, and ultimately improve their quality of life. Additionally, the platform serves as a remote communication platform for survivors of colorectal cancer and their families, as well as an educational platform for hospitals. The usability test results indicate that the platform performs well in terms of clinical usability, with excellent system quality, interface quality, and information quality. Learnability, usability, and satisfaction all reached a “good” level. Furthermore, the study found that the scores of usability dimensions were not related to gender, age, or educational level, suggesting that the middle-aged and older adult population (younger than 75 y old) may have potential in learning to use new platforms. Due to the limited sample size, the statistical power to detect and confidently estimate the effects of factors influencing usability is reduced, thereby potentially limiting the robustness and generalizability of the conclusions. Results from the unstructured interviews show that most users had a positive evaluation of the platform, noting its comprehensive and professional content, practical multimedia functions, ease of operation, and interesting and innovative features. However, issues with slow loading and complex operation were identified in terms of platform usability.

Our study represents the first application of metaverse technology, specifically in the family rehabilitation of survivors of colorectal cancer, offering new research perspectives in this field and providing insights for other cancer studies. We obtained a series of meaningful results with important implications for future medical practice and research.

### Platform Usability and Influencing Factors

The scores for system quality, information quality, and interaction quality are relatively high, indicating a strong correlation between these performance aspects and the quality of content provided by the platform, including convenience, clarity, and interactivity. This suggests that the platform’s content design is relatively excellent. However, the score for learnability is the lowest, implying that users may find it challenging to grasp the platform’s functions and operations during initial use, resulting in a higher learning cost and time investment. Effectiveness is above the passing line, indicating that the platform may not effectively assist users in achieving their goals quickly and accurately, requiring additional effort. Satisfaction is at a moderate level, possibly influenced by software operations.

Violin plot analysis reveals that while the learnability score is concentrated around the passing line (50 points), scores for other dimensions exhibit 2 concentrated distribution areas, both above the passing line. The high-score concentration area reflects that most users perceive the platform’s performance as good (except for learnability), further confirming the platform’s excellent performance in terms of usability. However, attention should still be paid to the platform’s learnability.

This study observed that patient groups rated platform usability lower than family members. Usability fundamentally reflects the degree of human-machine adaptation in user interaction interfaces. It is worth noting that this evaluation difference may be closely related to the physiological and psychological characteristics of the evaluators. Most patients are in the treatment period (chemotherapy and ostomy), with certain physiological and psychological issues, which may have multiple impacts on evaluation results. First, treatment-related complications (such as neuropathic pain and cancer-related fatigue) may affect patients’ cognitive function and technical operational abilities, thereby reducing their acceptance of technology. Second, the anxiety and depression commonly found in patients during the treatment period may influence the objectivity of evaluations, leading to a negative evaluation bias. Additionally, treatment-related physical limitations (such as peripheral neuropathy and decreased visual acuity) may directly impact the platform’s utility, lowering patients’ acceptance of the technology. When promoting clinical application in the future, a simple assessment of patients’ physical conditions should be conducted, and based on the patients’ situation, the platform’s target users could be patients or family members, with family members learning the platform content and then supervising patients to complete intervention tasks, which could be a better choice.

There were no significant differences in scores between genders across dimensions. Differences in scores across dimensions were observed by age, with the 18‐ to 44-year age group generally scoring higher than those aged 45 years and older in software operation–related ratings (learnability and effectiveness), and those with a college education or higher scoring higher than those with other educational levels. In terms of software quality, the older than 60 age group scored the lowest, while the junior high school and below education group scored lower compared with the relatively higher educated group. This may be related to the characteristics of these 2 groups, as individuals with lower education levels and older individuals may prefer a simple, visually engaging, and easy-to-understand platform compared with other groups. Radar chart analysis shows that the scores across platform dimensions are uneven, especially with weaknesses in learnability and effectiveness, indicating the need for improvement.

Although there are score differences in age and educational level distributions, they have not reached statistical significance. This finding may suggest that the rehabilitation platform may be universal for individuals aged 18‐75 years, providing similar levels of service and support to different user groups. Particularly noteworthy is that age has not become a limiting factor in learning new platforms, providing more possibilities for older adults to embrace new technologies. This result differs from common perceptions. While some older adult individuals may encounter operational issues during actual use, there are also older adult individuals, especially those with higher education levels, who can easily use the Metaverse platform.

Technical experience may influence users’ attitudes toward the Metaverse platform [33]. During the experiment, it was found that some young people also encountered difficulties in using the platform. These young people are usually more educated but may lack experience with online games and are predominantly female. On the other hand, some users, despite having lower levels of education, were able to use the Metaverse platform smoothly and showed higher satisfaction. These users typically have more experience with online games.

Dependence on network speed also affects the usability of the Metaverse platform. In areas with slow network speeds, users may encounter issues, such as slow loading and lagging. Additionally, the mobile phone system can also affect the user experience of the Metaverse, as older versions of mobile phone systems may not fully support Metaverse apps. Through communication with the technical team, these issues can be resolved. This is also one of the reasons for user dissatisfaction. However, with the continuous advancement of technology, issues resulting from low network speeds and operating system configurations will gradually be resolved, leading to a continuous improvement in user satisfaction with the Metaverse platform.

During the experiment, it was observed that users’ initial impressions and attitudes toward the Metaverse platform also influence their enthusiasm and effectiveness in learning to operate the platform. The more positive the attitude, the easier the learning process, and users are more likely to master the platform. For those who initially believe they cannot grasp the Metaverse platform, they often encounter difficulties in practical operation. As long as they maintain a curiosity for learning, many older adult individuals can also easily get started. Therefore, the focus should be on “technological resistance.” When facing “technology,” do not be intimidated by it; instead, embrace it and believe in your ability to handle technology. Technology exists to serve humanity, making life more convenient and enjoyable. If a technology makes users feel difficult, it will eventually be eliminated because it does not align with the original intention of technological development. When faced with a new technological platform, maintain an open mindset and bravely try it out. Therefore, it is important to value users’ initial feelings and attitudes toward the platform, as this will directly impact the degree of user acceptance and the effectiveness of use of the Metaverse platform. It is recommended that when promoting the Metaverse platform, particular attention should be given to users’ psychological attitudes and emotional feedback, and through active guidance and training, help users develop a positive perception of the platform, thereby enhancing their learning and operational effectiveness, and ultimately improving the overall user experience and satisfaction.

Due to the limited research sample and insufficient demographic variables, this study has not fully explored the factors influencing the use of the Metaverse platform, which may involve cultural levels, gender, familiarity with online games, self-efficacy, and so on. In conclusion, although the Metaverse platform holds great potential in the field of rehabilitation services, there are still some limiting factors that need to be addressed, such as the impact of user group characteristics on platform use, network speed, and mobile system compatibility issues. With technological advancements and continuous platform optimization, it is believed that these limiting factors will gradually be resolved, leading to a continuous improvement in user satisfaction and popularity of the Metaverse platform, providing a better rehabilitation service experience for more users.

### Platform Advantages and Clinical Benefits

The metaverse platform, as an emerging rehabilitation tool, has demonstrated unique advantages and potential in the field of remote rehabilitation. Answers to open-ended questions indicate that users generally give positive feedback on the platform content, considering it practical, interesting, comprehensive, instructive, clear, and easy to understand. The diversity and instructiveness of the rehabilitation content enable users to better understand and absorb information, thereby enhancing the effectiveness of rehabilitation. However, issues such as the complexity of platform operation, potential lag during operation, and lack of interactivity affect user experience and platform learnability. Overall, while the platform content can adequately meet clinical needs and is well-structured, there are still some areas in operational aspects that require improvement.

The diverse intervention content and methods provided by the metaverse platform offer users a comprehensive rehabilitation service experience. First, the platform offers a wealth of graphic and video resources, providing users with multiple sensory input pathways that aid in information comprehension and absorption. Compared with plain text, videos and graphics are more vivid and intuitive, conveying information more effectively, making it easier for users to understand and remember, thus enhancing intervention effectiveness and sustainability. Second, the metaverse platform incorporates questionnaire functions to help survivors assess whether they have achieved their set goals. This personalized assessment method allows users to gain a clearer understanding of their rehabilitation progress, helping them adjust their rehabilitation plans and improve rehabilitation outcomes. Additionally, the platform introduces gamification mechanisms, including setting tasks and providing rewards, which motivate survivors during the rehabilitation process, enhancing their motivation and sense of involvement. Furthermore, the metaverse platform provides auxiliary design features to help users better receive information, such as the function of automatically reading text on images by clicking on them, digital human navigation services, and signposts, greatly facilitating the acceptance of intervention information by different age groups, enhancing intervention universality and operability. These advantages collectively make the metaverse platform the preferred choice in the field of remote rehabilitation.

Moreover, the metaverse multidimensional rehabilitation platform also offers social interaction and support systems, allowing users to interact and communicate in real time with other survivors or health experts. This social support can increase users’ motivation for rehabilitation and self-management capabilities, promote information sharing and experience exchange, and build a supportive rehabilitation community [34]. Additionally, the platform has cross-platform and cross-device compatibility, allowing users to access the platform anytime, anywhere through different devices, achieving seamless connection of the rehabilitation process from the hospital to the home, providing users with a convenient and smooth rehabilitation experience.

Research results indicate that the metaverse platform has been successful in meeting user needs, providing effective information, and offering a good user experience, demonstrating the potential to provide excellent rehabilitation support for survivors of colorectal cancer and their families. Research on the effectiveness of metaverse rehabilitation therapy for cerebral palsy has shown significant improvements in various health parameters compared with traditional physical rehabilitation treatments [35]. Experts have pointed out that metaverse rehabilitation exercises can help patients with stroke improve cognitive function, attention, muscle endurance, cardiorespiratory function, maintain appropriate weight, and prevent stroke recurrence [36]. Floreo, a company in the United States, created the first behavioral therapy metaverse, teaching social, behavioral, communication, and life skills to patients with autism spectrum disorders, attention-deficit/hyperactivity disorder, anxiety disorders, and other neurodiversity disorders. Additionally, the metaverse is used to build communication platforms for patients with cancer and their families, assisting patients in improving the discomfort caused by illness and enhancing their social adaptability [19]. These applications emphasize the potential advantages of incorporating metaverse-based approaches into patient rehabilitation interventions and disease management. The metaverse holds promise for bringing new opportunities to rehabilitation medicine, improving the quality of life of survivors of colorectal cancer, and reducing the incidence of complications.

The metaverse has the potential to shorten social distances, facilitate simultaneous online learning for multiple users, and stimulate participants’ rehabilitation motivation. Competitive incentives and mutual encouragement among participants can help in task completion. The research team of Mizuta et al [37] reported that compared with simply posting exercise videos on YouTube (Google), providing exercise videos through the metaverse platform can increase physical activity among young individuals with similar exercise motivations, while reducing feelings of isolation during exercise. Despite similar video content, the viewing experience provided by the metaverse is more engaging and immersive, adding challenge and enjoyment. The metaverse also offers additional benefits, such as avoiding the embarrassment of visiting clinics and increasing the acceptance among patients who find it inconvenient to seek treatment at hospitals due to psychological barriers. Participants tend to label metaverse-based therapeutic content as entertaining, enjoyable, or convenient [33].

The metaverse holds great potential in the health care field. Simulation programs for early-onset schizophrenia care based on the metaverse can serve as an alternative for high-quality care education, replacing clinical psychiatric practice without spatial constraints [38]. The application of the metaverse in medical training programs has shown significant effects. Using the metaverse in onboarding training and nurse and resident training programs has led to improvements in participants’ knowledge, skills, and confidence [39]. The application of the metaverse in clinical handover nurse training has significantly improved nurses’ self-efficacy, handover skills, and satisfaction [40]. Overall, the metaverse has shown good effectiveness in the education and training fields, indirectly confirming its clinical feasibility, given that the metaverse multidimensional rehabilitation platform mainly focuses on health education. While the application of the metaverse in the rehabilitation field is limited, existing clinical experiences demonstrate significant potential, such as virtual physical therapy to guide rehabilitation patients in exercises and movements [41].

Although the metaverse brings many benefits, the current multidimensional rehabilitation platform still has some limitations, including insufficient content, complexity of operation, lack of interactivity, inadequate professional services, lack of prompts, unclear information, and difficulty in screening. To address these issues, the team has further optimized the metaverse platform, including enlarging operation buttons and changing the taskbar to prominent patterns. Additionally, content directories have been added at the entrance of each room to help users find the desired content. Outstanding issues include professional services and interactivity, mainly because the platform has not been formally deployed in clinical practice, team members have not fully engaged with the platform, and professional services and interactive content have not been fully explored. This limitation is expected to be partially addressed in formal clinical studies.

In conclusion, the metaverse platform, as an innovative rehabilitation tool, has shown tremendous potential and advantages, providing innovative solutions for the rehabilitation of survivors of colorectal cancer. Its diverse intervention content and social support system offer users a comprehensive and effective rehabilitation service experience, with the potential to enhance survivors’ quality of life. However, operational aspects still require optimization, with simplification being a key focus for future improvements. Through continuous optimization and enhancement, the metaverse platform is poised to become a leading choice in the field of rehabilitation, enhancing survivors’ quality of life and rehabilitation outcomes.

### Limitations

This study has the following limitations. First, the sample size is small, with only 18 participants, which limits our ability to conduct detailed analyses of different participant groups and prevents an in-depth exploration of multiple potential factors affecting satisfaction with the metaverse. This also limits the statistical testing power of the results and the generalizability of the conclusions, especially when facing diverse user needs, where the representativeness of the results may be affected. Although the sample size in this study exceeds the minimum sample size recommended by the US Clinical Trial Registration Platform for usability studies (fewer than 10 people), it is still difficult to completely exclude the risk of bias caused by insufficient samples. Second, the assessment tools used in this study are mainly self-report scales, which are subjective to a certain extent. Participants’ responses may be influenced by social desirability bias, memory bias, or personal emotional states, thereby introducing response bias or measurement errors that affect the objectivity and accuracy of the data. Third, there are significant individual differences in users’ preferences for platform content. For example, some patients are more concerned with mental health education content, while others place more emphasis on content related to physical activities. This heterogeneity in content preferences may lead to large variations in evaluations of usability indicators, affecting the internal consistency and generalizability of the results. In addition, the data in this study are mainly based on short-term usage feedback, lacking longitudinal observation of the platform’s long-term usability and long-term user satisfaction, making it difficult to reflect the dynamic changes in user experience and long-term effects. Finally, the study lacks objective behavioral data support (such as usage duration and interaction frequency) and mainly relies on subjective evaluations. Future research should combine more objective indicators to comprehensively and multidimensionally assess the platform’s usability and user experience. In summary, future studies should expand sample sizes, cover a broader range of population characteristics, adopt mixed research methods that combine subjective evaluations with objective data, and conduct long-term follow-up observations to comprehensively and in-depth investigate factors influencing the usability of metaverse platforms, thereby enhancing the scientific rigor and practical guidance value of the research.

### Conclusions

This study demonstrates the potential and prospects of metaverse technology in colorectal cancer rehabilitation, providing personalized and convenient rehabilitation services for patients and their families with the aim of improving patient quality of life and rehabilitation outcomes. However, integrating metaverse technology into clinical practice still presents challenges. In the future, exploring the combination of virtual reality, augmented reality, and other technologies to provide comprehensive, personalized rehabilitation platforms and integrating AI to achieve more intelligent and precise medical services could be beneficial. Emphasis should be placed on safety and privacy protection to ensure compliance with regulations and to safeguard patient privacy and data security. Additionally, integrating metaverse technology with traditional medical models to achieve complementary advantages and to build a more complete medical ecosystem can provide patients with comprehensive and continuous medical services. Importantly, this study is the first to apply metaverse technology to the family rehabilitation of survivors of colorectal cancer, bringing new ideas and opportunities to related research and practices. Our findings not only offer innovative rehabilitation support for survivors of colorectal cancer and their families but also provide insights and inspiration for other cancer rehabilitation research. This metaverse-based rehabilitation platform has the potential to improve the quality of life of patients and their families and may offer medical institutions more effective rehabilitation services and educational outreach avenues.

Although the application prospects of metaverse technology in the medical field are broad, continuous exploration and improvement in clinical practice are necessary to achieve better medical outcomes and service quality. Through ongoing innovation and collaboration, metaverse technology has the potential to bring breakthroughs and advancements to the medical field, benefiting more patients and their families.

## Supplementary material

10.2196/63543 Multimedia Appendix 1 Additional tables and figures.

## Abbreviations

AI: artificial intelligence
ICF: the International Classification of Functioning, Disability, and Health
InfoQual: Information Quality
IntQual: and Interaction Quality
mHealth: mobile health
PSSUQ: Post-Study System Usability Questionnaire
SUS: System Usability Scale
SysQual: System Quality
