# Supplementary material for: Investigating the Usability of a Multidimensional Metaverse Rehabilitation Platform for Survivors of Colorectal Cancer: Mixed Research Design Experiment
Source: JMIR Serious Games. 2026 Jul 31;14:e63543. doi: 10.2196/63543 (PMC13426120; doi:10.2196/63543)
Supplement: Multimedia Appendix 1 [file games-v14-e63543-s001.docx]

**Multimedia Appendix 1**


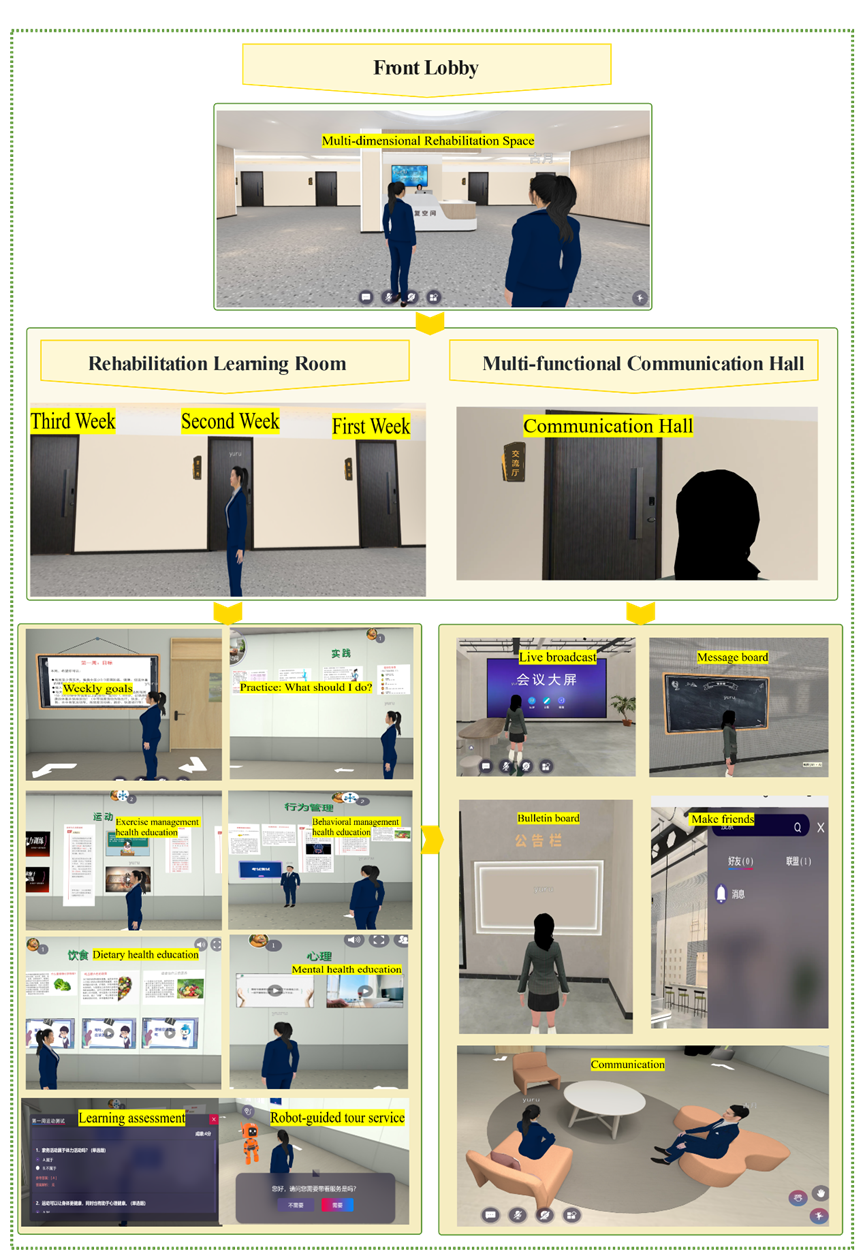


**Multimedia Appendix 2**

During the optimization phase of the platform, a professional optimization team was formed, consisting of 7 members. The specific information is as follows:

**Composition of the Optimization Team**

| No. | Gender | Age | Highest Education | Role |
| --- | --- | --- | --- | --- |
| 1 | Female | 50 | PhD | Nursing Teacher |
| 2 | Female | 42 | Master's | Clinical Nurse |
| 3 | Male | 40 | PhD | Clinical Physician |
| 4 | Female | 36 | Master's | Clinical Nurse |
| 5 | Female | 30 | Bachelor's | Rehabilitation Therapist |
| 6 | Female | 35 | Master's | CRC Survivor |
| 7 | Male | 31 | Bachelor's | CRC Survivor |

**Multimedia Appendix 3**

The specific intervention content of the platform is as follows:

Week 1 Content Design

|  | Text + images | Video | Questionnaire |
| --- | --- | --- | --- |
| Target | 1. Goals for the first week  2. Welcome Message (Voice) |  |  |
| Practice | 1. How to consume plant-based foods  2. What are the plant-based foods?  3. How to exercise  4. How to make exercise more comfortable  5. How much protein and energy to consume  6. Sources of protein |  |  |
| Knowledge sharing (diet and nutrition, exercise, psychological interventions, behaviour management) | 1. Healthy Eating Essentials  2. Introduction to Colorectal Cancer  3. Colorectal cancer symptoms and signs  4. Colorectal cancer risk factors  5. Prevention of colorectal cancer  6. Stress management strategies  7. Coping with fear of recurrence  8. Dietary fiber  9. Sources of dietary fiber  10. Carbohydrate sources  11. Energy content of each food  12. Energy content of food  13. Energy content of fruits  14. The importance of exercise  15. Precautions for Exercise  16. Aerobic exercise  17. Integrate movement  18. How activity intensity is measured | 1. Interpretation of Dietary Guidelines for Chinese Residents (Self-made)  2. Introduction to Aerobic Exercise (Internet)  3. Baduanjin (Internet)  4. Muscle Stretch (Homemade)  5. Upper Limb Resistance Training (Homemade)  6. Lower Limb & Trunk Resistance Training (Homemade) | 3 sets of test questions; 1 set of questionnaires |
| Total | 26 | 6 | 4 |

Week 2 Content Design

|  | Text + images | Video | Questionnaire |
| --- | --- | --- | --- |
| Target | 1. Goal for the second week |  |  |
| Practice | 1. Eat colorful vegetables  2. How to choose vegetables  3. How to do resistance training |  |  |
| Knowledge sharing (diet and nutrition, exercise, psychological interventions, behaviour management) | 1. Quit smoking  2. Benefits of quitting smoking  3. How to stay smoke-free  4. Overcome the urge to smoke  5. The dangers of alcohol  6. Relaxation techniques  7. Stress reduction techniques  8. Nutrition after cancer treatment  9. Colorful vegetables  10. Phytochemicals  11. Vitamin D  12. Sleep Disorder Exercise Program  13. Exercise program for fecal disorders | 1. Smoking cessation video  2. How to eat constipation  3. How to eat nausea  4. How to eat diarrhea  5. How to eat vomiting  6. Exercise for cancer-related pain  7. Exercise for cancer-induced fatigue | 1 set of questionnaires |
| Total | 16 | 7 | 1 |

Week 3 Content Design

|  | Text + images | Video | | Questionnaire |
| --- | --- | --- | --- | --- |
| Target | 1. Week 3 goals |  | |  |
| Practice | 1. Try brand new cereals every day  2. How to exercise better  3. Exercise recommendations |  | |  |
| Knowledge sharing (diet and nutrition, exercise, psychological interventions, behaviour management) | 1. Benefits of whole grains  3. Core training  4. Bridge movement | 1. Colorectal cancer information  2. Get regular sleep  3. Relaxation therapy  4. Emotional catharsis  5. How to eat with a change in taste  6. How to eat when tired  7. Loss of appetite, weight loss, malnutrition diet  8. How to eat for weight gain  9. Pelvic floor rehabilitation  10. Cervical spine exercises | | 1 set of questionnaires |
| Total | 7 | 10 | 1 | |

Week 4 Content Design

|  | Text + images | Video | Questionnaire |
| --- | --- | --- | --- |
| Target | Week 4 goals； |  |  |
| Practice | 1. Reduce red meat intake  2. Plant-based protein  3. Ostomy care  4. Household changes | Ostomy care education | 1 set of questionnaires |
| Total | 4 | 1 | 1 |
